# Enteric Viruses and Fecal Bacteria Indicators to Assess Groundwater Quality and Suitability for Irrigation

**DOI:** 10.3390/ijerph14060558

**Published:** 2017-05-24

**Authors:** Osvalda De Giglio, Giuseppina Caggiano, Francesco Bagordo, Giovanna Barbuti, Silvia Brigida, Federica Lugoli, Tiziana Grassi, Giuseppina La Rosa, Luca Lucentini, Vito Felice Uricchio, Antonella De Donno, Maria Teresa Montagna

**Affiliations:** 1 Department of Biomedical Science and Human Oncology, University of Bari Aldo Moro, Piazza G. Cesare 11, 70124 Bari, Italy; giuseppina.caggiano@uniba.it (G.C.); giovanna.barbuti@uniba.it (G.B.); mariateresa.montagna@uniba.it (M.T.M.); 2 Department of Biological and Environmental Sciences and Technologies, University of Salento, Via per Monteroni, Campus Ecotekne, Monteroni di Lecce, 73047 Lecce, Italy; francesco.bagordo@unisalento.it (F.B.); federicalugoli@tiscali.it (F.L.); tiziana.grassi@unisalento.it (T.G.); antonella.dedonno@unisalento.it (A.D.D.); 3 Water Research Institute, National Research Council, Viale Francesco de Blasio 5, 70132 Bari, Italy; silvia.brigida@ba.irsa.cnr.it (S.B.); vito.uricchio@ba.irsa.cnr.it (V.F.U.); 4 National Institute of Health, Department of Environment and Health, Viale Regina Elena 299, 00100 Rome, Italy; giuseppina.larosa@iss.it (G.L.R.); luca.lucentini@iss.it (L.L.)

**Keywords:** groundwater pollution, irrigation, wells, Norovirus, Rotavirus, Enterovirus

## Abstract

According to Italian Ministerial Decree No. 185 of 12 June 2003, water is considered suitable for irrigation if levels of fecal bacteria (i.e., *Escherichia coli* and *Salmonella*) are within certain parameters. The detection of other microorganisms is not required. The aim of this study is to determine the bacteriological quality of groundwater used for irrigation and the occurrence of enteric viruses (Norovirus, Enterovirus, Rotavirus, Hepatovirus A), and to compare the presence of viruses with the fecal bacteria indicators. A total of 182 wells was analyzed. Widespread fecal contamination of Apulian aquifers was detected (141 wells; 77.5%) by the presence of fecal bacteria (i.e., *E. coli*, *Salmonella*, total coliforms, and enterococci). Considering bacteria included in Ministerial Decree No. 185, the water from 35 (19.2%) wells was unsuitable for irrigation purposes. Among 147 wells with water considered suitable, Norovirus, Rotavirus, and Enterovirus were detected in 23 (15.6%) wells. No Hepatovirus A was isolated. Consequently, 58 wells (31.9%) posed a potential infectious risk for irrigation use. This study revealed the inadequacy of fecal bacteria indicators to predict the occurrence of viruses in groundwater and it is the first in Italy to describe the presence of human rotaviruses in well water used for irrigation.

## 1. Introduction

In the last few years, many waterborne diseases from contaminated groundwater have been reported in countries at all levels of economic development, with outbreaks that result in large socioeconomic impacts [1,2,3]. As with other aquatic matrices, groundwater can be contaminated by microorganisms that originate from human activities (urban, industrial, and agricultural) and are released into the environment through direct discharge, insufficiently treated wastewater, leaking sewage, and septic systems [4,5,6,7,8,9,10,11,12,13,14]. For these reasons, groundwater quality becomes extremely important for human consumption or irrigation purpose.

Agriculture is a very profitable sector in some countries. During pre-harvest cultivation, there are various routes of contamination, which usually include application of organic wastes as fertilizer, contamination of water used for irrigation with fecal material, contact with inadequately-treated sewage or sewage-polluted water and direct contamination by livestock [15,16].

Presently, there is increasing evidence of the contamination of foodstuffs by irrigation water and an association of foodborne outbreaks with contaminated vegetables, juices, and other products [17]. Therefore, water quality needs to be monitored for the irrigation of cropland and for product health quality, especially that of fruits and vegetables that are intended to be eaten raw. A variety of pathogens can be present in water used for irrigation, including human-specific pathogens such as *Shigella*, Norovirus, hepatitis A virus, and zoonotic pathogens such as verocytotoxin-producing *Escherichia coli*, *Salmonella*, and *Cryptosporidium*, which primarily infect and replicate in the gastrointestinal tract of the host [17,18]. Enteric viruses are a public health concern because they are present in extremely high numbers in the feces of infected individuals. In addition, these viruses have a very low infectious dose and a strong ability to contaminate a great variety of environmental compartments because they are resistant to adverse conditions and many types of water treatment [19]. Some authors [20] have reported that the presence of fecal bacteria indicators (e.g., enterococci and fecal coliform bacteria) is not correlated with the occurrence of enteric viruses in water environments. In some cases, human enteric viruses have been found in aquatic ecosystems [21] and at dangerously high concentrations despite bacterial indicator data that are within the recommended limits [13].

Recently, the European Food Safety Authority began to closely examine the public health risks posed by pathogens, including viruses, that can contaminate foods of non-animal origin, with a special focus on leafy vegetables that are likely to be consumed raw [22]. Similar to other European countries, clean or potable water must be used for food production in Italy whenever possible, to prevent contamination of fresh produce during primary production [23]. In addition, parametric values for treated wastewater that is reclaimed for agricultural purposes in Italy, including primary food production, are established by Ministerial Decree No. 185 of 12 June 2003 [24]. According to DM 185/03, water is considered usable if it is within the following parameters for microorganisms of fecal origin: *E. coli* < 100 cfu/100 mL and *Salmonella* spp. absent from 1000 mL of water. Testing for the presence of other microorganisms (e.g., enterococci and enteric viruses) is not required. However, fecal bacteria in water sources such as surface waters and groundwater are not correlated to pathogens in previous studies, even when these pathogens are endemic in the region [11]. Consequently, a global indicator system for monitoring water quality, mainly for the detection of enteric viruses, is required. However, the detection and quantification of these viruses in aquatic environments is technically challenging, costly, and requires a great deal of time.

This study is part of more extensive research aimed at assessing the microbiological pollution levels of groundwater [12,25]. In this study, the main goals are: (i) to determine the bacteriological quality of groundwater for irrigation, as a function of both aquifer type and season; (ii) to measure the occurrence of the most common enteric viruses worldwide detected in water environments; and (iii) to compare the presence of these viruses with the standards for fecal bacteria outlined in DM 185/03.

## 2. Materials and Methods

### 2.1. Study Area

**Figure 1:**
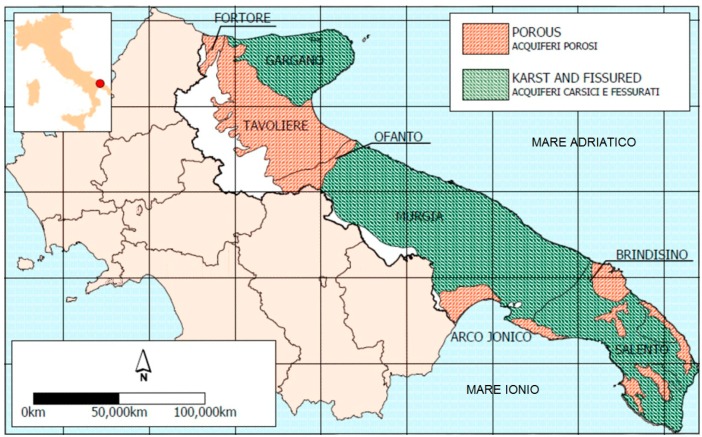
Distribution of aquifers in Apulia, southern Italy [25].

Apulia is a region in southeastern Italy that covers about 20,000 km^2^, with 4 million inhabitants. Thanks to its typical Mediterranean climate with mild and dry winters, hot summers, and irregular annual rainfall, the agriculture industry is widespread throughout a large part of this region. Agriculture has a prominent role in the economic context of the region, which ranks second in Italy for the production of several foods, particularly fresh fruit and vegetables to be eaten raw (e.g., salad greens, tomato, fennel, and celery). The agricultural activity in this area is very intensive and requires a large amount of water for irrigation purposes. Because of the geographical features and hydrogeological conditions (mostly karst-fissured) in Apulia, as well as the absence of significant rivers or lakes, groundwater covers 75% of irrigation demand for the local population [26]. The groundwater wells are supplied by the Gargano, Murgia, and Salento karst-fissured aquifers (up to 400 m deep) and by the Tavoliere, Piana brindisina, and Arco Jonico Tarantino Occidentale (Arco Jonico) porous aquifers (less than 60 m deep) (Figure 1) [25].

### 2.2. Water Sampling

**Figure 2:**
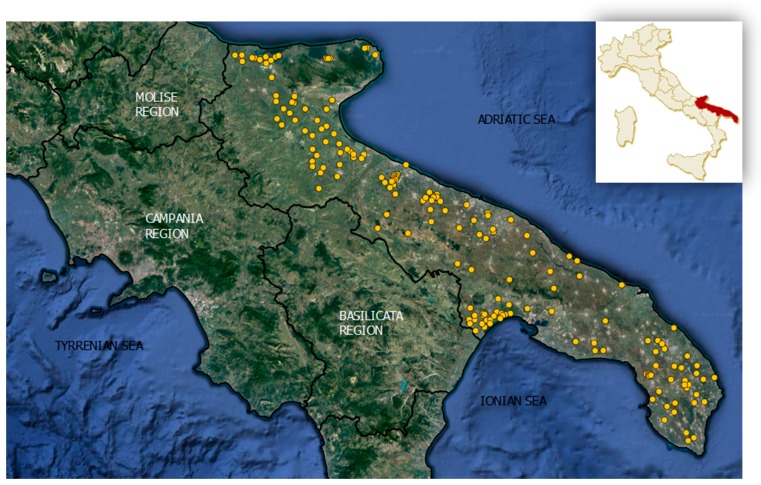
Distribution of sampled wells in Apulia, Italy.

In 2014, 182 wells used for irrigation were sampled twice, in winter and in summer (in January and in July, respectively), yielding a total of 364 samples (Figure 2).

Data on the rainfall (mm) and the mean temperature (T °C) recorded in the area of study were acquired and elaborated by the Regional Environmental Protection Agency [27].

Following the procedure of De Giglio et al. [25], water samples were collected—after flushing for 10 min—in sterile containers between 9:00 a.m. and 12:00 p.m., under calm atmospheric conditions, with no rain, and were transported into a refrigerator (+4 °C) and processed for 5 h. Overall, 182 wells draw groundwater from two different hydrogeological settings: 236 samples were from Mesozoic karst-fissured aquifers located in the areas of Gargano (GA, *n* = 28), Salento (SA, *n* = 82), and Murgia (MU, *n* = 126); and 128 samples were collected from Quaternary porous aquifers that represent the hydrogeological settings of Arco Jonico (JON, *n* = 40) and Tavoliere (TAV, *n* = 88).

### 2.3. Bacteria Detection

Two liters of water were collected from each well and used to test for the following bacteria: *E. coli* and total coliform bacteria, *Salmonella* spp., and enterococci.

*E. coli* and total coliform. A 100 mL aliquot of the water sample was filtered through a cellulose ester membrane (47 mm Ø and 0.45 μm-pore size; Millipore, Milan, Italy), placed on Tergitol 7-Triphenyl tetrazolium chloride agar (Biolife Italiana srl, Milan, Italy) and incubated at 36 ± 2 °C for 24 ± 2 h. If no typical colonies were present, the samples were incubated for an additional 24 ± 2 h. Lactose-positive colonies were subcultured onto a tryptone tryptophan medium (Sigma-Aldrich, St. Louis, MO, USA) and incubated at 37 ± 1 °C for 24 ± 2 h. If the resulting colonies were oxidase negative and indole positive, then they were assumed to be *E. coli*, while if they were oxidase negative, they were assumed to be total coliform [28].

*Salmonella* spp. A 1 L subsample of each water sample was filtered by a cellulose ester membrane (142 mm Ø and 0.45 μm pore size; Millipore, Milan, Italy). This membrane was placed in 100 mL sterile 0.1% (w/v) peptone water (Thermo Scientific Oxoid, Milan, Italy) and homogenized for 1 min. Subsequently, an aliquot of the homogenized material was mixed with a selective enrichment medium, consisting of selenite cystine broth (Biolife Italiana srl, Milan, Italy). After incubation for 24 h at 35 °C, it was subcultured on two agar plates, brilliant green and xylose lysine deoxycholate (Becton Dickinson, Heidelberg, Germany), and incubated for another 24 h at 35 °C. From each plate, at least one colony suspected of being *Salmonella* was inoculated on triple sugar iron and lysine iron agar (Biolife Italiana srl, Milan, Italy), incubated for 24 h at 35 °C, and typed via specific serological tests [29].

Enterococci. A 100-mL aliquot of each water sample was filtered through a cellulose ester membrane (47 mm Ø and 0.45 μm pore size; Millipore, Milan, Italy). The membrane was placed over a Slanetz and Bartley agar medium (Biolife Italiana srl, Milan, Italy) and incubated at 36 ± 1 °C for 48 h. The colonies ranged in color from pink to dark red and brown, but only catalase and esculin hydrolysis positive ones were considered to be enterococci [30].

Water was determined to be unsuitable for irrigation use if levels for at least one microorganism of fecal origin exceeded the DM 185/03 limits in at least one of the two collected samples.

### 2.4. Enteric Virus Detection

Twenty liters of water were collected from each well, to test for the presence of Norovirus, Enterovirus, Rotavirus, and Hepatovirus A by molecular methods.

#### 2.4.1. Concentration Process

The 20-L water samples were concentrated by the tangential flow ultrafiltration technique using polypropylene membranes with a 10 kDa molecular weight cut-off and a pore size of 0.001 to 0.01 microns, in two consecutive steps: (a) using a MAXIFLEX 25 ECO filtration system (Schleicher &Schuell, Dassel, Germany) equipped with a polysulphone membrane with surface area 0.1 m^2^; (b) using an ULTRAN-MINIFLEX filtration system (Schleicher & Schuell) equipped with a smaller membrane (0.0024 m^2^). Before performing these steps, the filtering membranes of both systems were conditioned with 200 mL (first step) and 20 mL (second step) of 3% beef extract at pH 7. The concentration process in both steps was completed by washing the filters with 200 mL and 20 mL of 3% beef extract at pH 9, respectively, to remove any viral particles that may have adhered to them. A final volume of about 300 mL was obtained after the first concentration and about 40 mL after the second concentration; therefore, each water sample was concentrated from 20 L to 40 mL. After neutralization of the pH with 1 N HCl, the concentrated samples were decontaminated with chloroform (1:10 v/v) before testing using molecular biology techniques. The effectiveness of this method has been previously assessed by Grassi et al. [19].

#### 2.4.2. Viral Analysis

Viral RNA was extracted from 140 μL of concentrated groundwater samples using the QIAamp Viral RNA Mini Kit (QIAGEN AG, Basel, Switzerland), in accordance with the manufacturer’s instructions. The RNA was then reverse-transcribed and amplified by one-step real time- polymerase chain reaction (RT-PCR), with CFX 96 Touch Deep Well Real Time detection System (Bio-Rad, Hercules, CA, USA) and CFX Manager Software (Bio-Rad, Hercules, CA, USA), using kits specific for qualitative detection of Hepatovirus A (WHATfinder HAV ID assay, Generon, Modena, Italy), Norovirus I/II and Rotavirus (G-DiaNotaTM, Diagenode Diagnostics, Liège, Belgium), and Enterovirus (Human Enterovirus, Diagenode Diagnostics, Liège, Belgium), according to the manufacturer’s instructions.

Hepatovirus A. The kit contains primers and probe pre-blended master mix according to CEN/ISO 15216 Protocol [31]. PCRs were carried out according go fast thermal profile under cycling conditions of 50 °C for 15 min and 95 °C for 2 min followed by 45 cycles of 95 °C for 15 s, and 60 °C for 30 s.

Norovirus I/II and Rotavirus. G-DiaNotaTM contains RNA double-dye probe & primers to target genes: RdRp/Capsid junction, NKP2, NSP3. PCRs were carried out under cycling conditions of 50 °C for 30 min and 95 °C for 10 min followed by 45 cycles of 95 °C for 15 s, 55 °C for 30 s and 68 °C for 60 s.

Enterovirus. The kit contains RNA primers & double-dye probe to target genes: VP3, VP1, and 2A. PCRs were carried out under cycling conditions of 50 °C for 30 min and 95 °C for 10 min followed by 45 cycles of 95 °C for 30 s, 55 °C for 30 s and 68 °C for 30 s.

### 2.5. Statistical Analysis

The results of microbiological testing were entered into a database and statistically analyzed using MedCalc version 12.3 (MedCalc Software, Ostend, Belgium).

The frequency of positive samples was determined for all parameters. The lowest, the highest and the median concentration were also calculated for bacteriological parameters.

The possible normal distribution of values was determined for each of the five aquifers and the two sampling seasons (winter and summer).

A comparison of the frequency of positive samples in different groups was performed using the chi-squared test. Because the values determined by the analyses did not follow a normal distribution, a comparison of the bacterial indices was performed using the Mann-Whitney test to compare two groups and the Kruskal-Wallis test to compare more than two groups. Differences were considered statistically significant with *p*-values < 0.05.

## 3. Results

**Figure 3:**
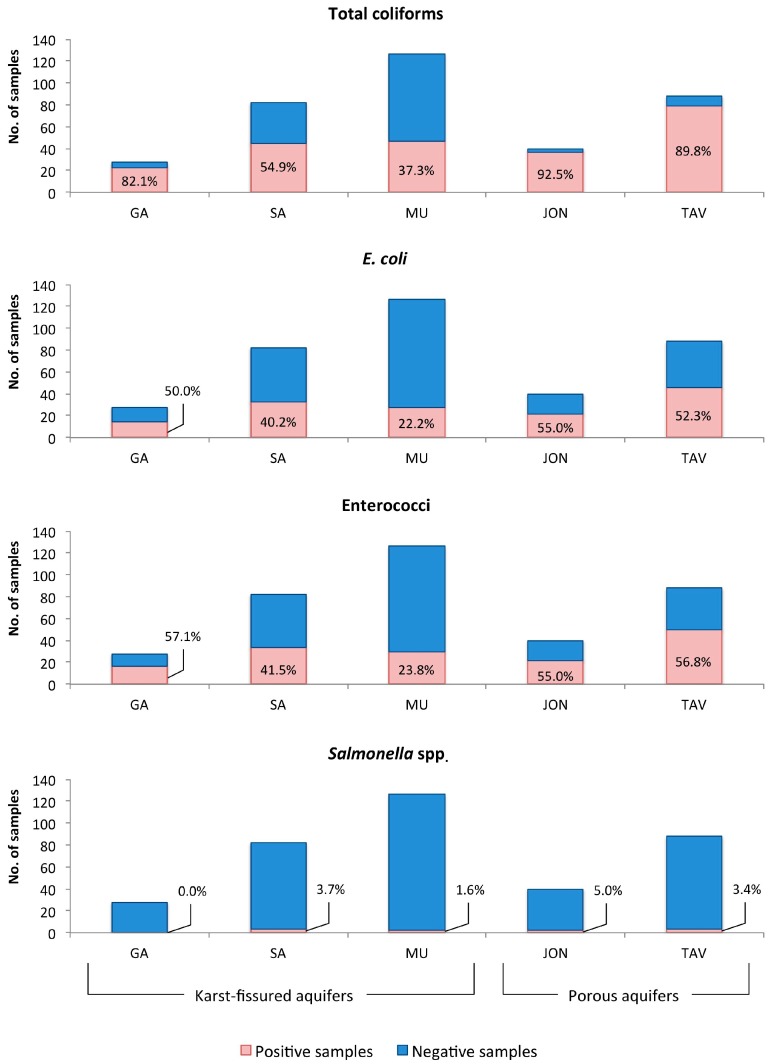
Positive samples (%) by testing of Apulian aquifers for bacteriological indicators. GA, Gargano; SA, Salento; MU, Murgia; JON, Arco Jonico; TAV, Tavoliere.

Indicator bacteria were found in 233 samples (64%) collected from 141 wells (77.5%). Significantly higher values (*p* < 0.05) were detected in shallow porous aquifers (90.6%; 92.5% in JON and 89.8% in TAV) than in deep karst-fissured aquifers (49.6%; 82.1% in GA to 38.9% in MU). Except for *Salmonella* spp., the frequency of indicator bacteria appeared to be significantly different (*p* < 0.05) among the sampled areas (Figure 3).

Quantitative data of the bacteriological parameters are reported in Table 1. Except for *Salmonella* spp., significantly different concentrations (*p* < 0.05) were recorded in the various aquifers for these indicator bacteria, with higher values for total coliforms in TAV (35 cfu/100 mL, respectively) and for enterococci in JON (3 cfu/100 mL).

With respect to enteric viruses, 27 (7.4%) of the 364 samples from 27 different wells tested positive, for at least one type of virus, 19 (8.1%) from karst-fissured aquifers and 8 (6.2%) from porous aquifers (Table 2); there was no significant difference between the two types of aquifer or spatially. Among the 27 samples that tested positive for at least one type of virus, 18 (66.7%) samples were positive for Noroviruses, 5 (18.5%) for Rotavirus, and 4 (15.8%) for Enterovirus. Hepatovirus A was not isolated.

According to DM 185/03 standards, 35 (19.2%) of the 182 examined wells were unsuitable for irrigation purposes owing to levels of *E. coli* higher than 100 cfu/100 mL and/or the presence of *Salmonella* spp. in 1 L of water. Among the 35 unsuitable wells, four were also positive for Norovirus or Rotavirus. Moreover, the presence of viruses was detected in 23 (15.6%) of the 147 wells determined to be suitable for irrigation purpose. Consequently, a total of 58 (31.9%) wells contained water that posed a potential infectious risk if used for crop irrigation, with a statistically significant increase (*p* < 0.05) of the number of unsuitable wells in both types of aquifer (Table 3).

The temporal trend of the bacteriological and virological parameters did not show a statistically significant difference between the two seasons (Table 4).

**Figure 4:**
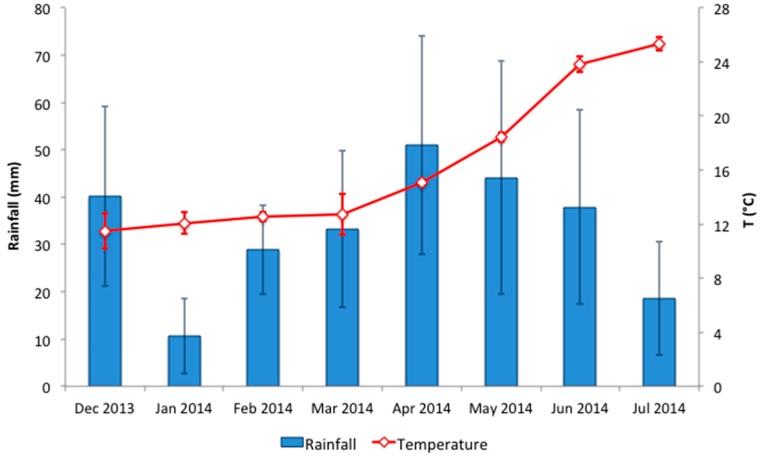
Mean (±standard deviation) monthly temperature and rainfall in Apulia, Italy (December 2013–July 2014).

Mean monthly temperature and rainfall in Apulia region from the month before the beginning of the sampling to the end of the sampling (Figure 4) revealed that the temperature ranged from 11.5 ± 1.3 °C in December 2013 to 25.4 ± 0.5 °C in July 2014 with significant difference (*p* < 0.05) between the two sampling periods. On the contrary, there was no significant difference (*p* > 0.05) in the rainfall during the pre-sampling periods. In particular, the months with lower rainfall were January 2014 with 10.6 ± 11.2 mm and July 2014 with 18.6 ± 15.9 mm, while the highest rainfall was in April 2014 with 51.0 ± 15.1 mm.

## 4. Discussion

The laws governing water quality in Italy allow for virus detection only for drinking water, when a sanitary risk is suspected by local health authorities. There is no explicit provision for virus testing of water used for irrigation in the regulations established for water used in primary production (i.e., Reg. (EC) No. 852/2004) or in the national DM 185/03 for reclaimed treated wastewater for agricultural use.

The consumption of raw vegetables is widespread in Apulia. Therefore, the microbiological quality of irrigation water is a public health concern because it can impact food quality during the pre-harvest stage. Among different types of irrigation waters [32], our study considered only the groundwater because it can be contaminated with surface runoff and with human and animal wastewater that represent a potential risk of infection when used as derived fertilizers, if not properly treated.

This study represents a large-scale microbial sampling campaign of which very few are reported in the peer reviewed literature [7,9,11]. Particularly, to the best of our knowledge, this study represents the first investigation of bacterial and viral contamination of irrigation groundwater in the Apulia region. To date, the data available for this region have been geographically and temporally fragmented and address viral and bacterial contamination separately [8,25].

Our study shows widespread fecal contamination of Apulian aquifers, owing to the presence of fecal indicator bacteria, including total coliforms, *E. coli*, enterococci, and *Salmonella*. These results represent a possible risk for primary production, especially in the case of fresh fruits and vegetables intended to be consumed raw. Assessment of this possible risk must be integrated with other data, including timing and method of irrigation, soil type, and contact of water with the edible portion of plants, among others.

According to other authors [18,33], in our study the presence of viruses was detected in irrigation waters determined to be suitable by considering only those bacterial indicators proposed by DM 185/03 (i.e., *E. coli* and *Salmonella*). Moreover, 19.2% of wells were determined to be unsuitable for irrigation purposes; however, if enteric viruses are included, this figure increased to 31.9%. This suggests that fecal bacteria cannot always be an adequate indicator for assessing virological safety of water used for irrigation or human consumption, highlighting the lack of specificity and sensitivity test [11]. Instead, enteric viruses are promising direct fecal pollution source tracking markers due to their prevalence in host feces and host specificity and to the easy mobility of the subsurface environment [34].

Among enteric viruses, Norovirus, Rotavirus, Enterovirus and Hepatovirus A have been selected in this study as they are the most common viral agents worldwide detected in water environments causing gastroenteritis through ingestion of contaminated water or fruit and vegetables [11,35,36,37]. Norovirus, Rotavirus, and Enterovirus were isolated in our study whereas Hepatovirus A virus was not.

In Italy, the presence of human Norovirus in sewage and water matrices impacted by sewage has been widely reported; moreover, seven waterborne Norovirus outbreaks have been described since 2000, which originated from sewage-polluted drinking water and recreational waters and involved more than 4000 cases [38]. Our research is the second in Italy reporting the presence of Norovirus in groundwater because to date only one study documented the presence of Norovirus GI and GII in 15.4% of samples collected during 2009 in the Latium region of central Italy [39].

Rotaviruses are the major cause of childhood diarrheal disease detected in different water environments worldwide, including groundwater [11,40], even though waterborne outbreaks have been only sporadically documented. In Italy, Rotaviruses are a major cause of hospitalization among pediatric-aged children in Apulia, especially in the Salento area [41]. These viruses have been detected in raw and treated sewage and surface water [19,42]; however, there has been little evidence of groundwater contamination [8]. The present study is thus the first in Italy to describe the presence of human Rotaviruses in wells used for irrigation.

Human Enteroviruses have been recovered worldwide from wastewater, surface water, groundwater, and finished drinking water, despite waterborne transmission having been infrequently confirmed. In Italy, Enteroviruses have been identified in groundwater samples by RT-PCR but have not been detected by cell culture isolation, suggesting inactive viral particles [36].

Hepatovirus A is the only enteric virus not detected in groundwater samples in the present study. Hepatitis A is a notifiable disease in Italy, which is considered to be of low/intermediate endemicity. Hepatitis A has been a serious public health problem in Apulia in recent years. Following a large epidemic in 1998, a vaccination program for toddlers and preadolescents was introduced in this region. Consequently, the incidence of disease has dramatically decreased between 2005 and 2014, which could explain our study results [43]. However, the increase of susceptible subjects and the permanence of environmental and behavioral risk factors may determine the onset of new epidemics in the case of re-entry of the pathogen, as Ajelli et al. [44] theorized through a mathematical predictive dynamic model.

Although we found evidence of viral contamination in the groundwater of Apulia and in wells considered suitable for irrigation, it is important to note that molecular detection may not accurately assess infectious viruses, resulting in overestimation of the occurrence of virus contamination and potential public health risks. Studies in which quantitative PCR (qPCR) has been conducted in parallel with cell culture isolation have revealed that a large proportion of positive qPCR tests were negative for infectious viruses by cell culture [45]. Some improvement could be realized by other techniques, in particular by combining PCR with a viability measurement, such us DNA pre-treatment, with the Propidium monoazide (PMA) [46].

Regarding the hydrogeological aspects of our findings, porous aquifers showed more frequent bacterial contamination than karst-fissured aquifers (90.6% vs. 49.6%, respectively). These results are not in agreement with other reports [6,47]; we believe the differences may be owing to well depths [4] and local characteristics, as reported in a previous study [25]. The areas with the highest levels of contamination are Arco Jonico (92.5%) and Tavoliere (89.8%). These aquifers are shallow and characterized by a succession with thickness of a few meters of permeable sandy—gravelly sediments intercalated by less permeable silt and clay layers, which facilitate microbiological contamination associated with intensive agriculture activities and seawater intrusion. By comparison, the karst-fissured aquifers flow through a Mesozoic carbonate succession; these are deeper and characterized by seasonal and climatic factors, mostly in the Gargano and Salento areas.

In our study, significant differences between porous and karst-fissured aquifers have not been demonstrated with respect to the presence of enteric viruses. Although soil type may influence the survival of these viruses, their survival is likely related to the degree of viral adsorption and their ability to travel hundreds of meters in the subsurface and remain viable [48,49]. Moreover, the penetration of pathogenic viruses to groundwater seems more likely than for pathogenic bacteria and protozoa owing to the extremely high numbers of enteric viruses shed into the environment and their resistance to common disinfection treatments [50]. These features make pathogenic viruses the most important candidates for fecal contamination of groundwater [51].

Our results show that microbial contamination seems to be uninfluenced by meteorological conditions (temperature and rainfall), although fecal bacteria concentrations in groundwater are well documented to rise after rainfall events in both karst [52] and alluvial sediment [9].

## 5. Conclusions

We found that fecal bacteria indicators were inadequate for predicting the occurrence of viruses in groundwater. Use of indicator bacteria to assess the hygienic quality of water is a widely debated issue. Presently, the investigation and the presence of viral pathogens in water sources remains at a stalemate, with possible negative impacts on public health. Water is used for various activities in primary production such as irrigation, preparing treatment solutions of plant protection products, humidification of produce, and post-harvest cooling and washing. Contact of potentially contaminated water with fresh produce and workers may pose a human health risk. According to international recommendations, good hygiene practices and basic risk assessment techniques should drive prevention, control, and mitigation of the risks of using water in the production of fresh fruits and vegetables.

## Figures and Tables

**Table 1:** Median concentration (range) of bacterial indicators in tested Apulian aquifers.

Bacteria	Karst-Fissured Aquifers			Porous Aquifers		Total	*p*-Value
	GA	SA	MU	JON	TAV		
*E. coli* ^a^	0.5 (0–120)	0 (0–3000)	0 (0–1700)	1 (0–1600)	1 (0–900)	0 (0–3000)	<0.001
*Salmonella* spp. ^b^	0 (0–0)	0 (0–120)	0 (0–20)	0 (0–8)	0 (0–7)	0 (0–120)	0.638
Total coliforms ^a^	30 (0–900)	2 (0–6000)	0 (0–2000)	24 (0–6000)	35 (0–2000)	10 (0–6000)	<0.001
Enterococci ^a^	2.5 (0–50)	0 (0–900)	0 (0–500)	3 (0–300)	1 (0–500)	0 (0–900)	<0.001

Note: Statistical significance calculated by Kruskal-Wallis test. ^a^ Unit is cfu/100 mL; ^b^ Unit is cfu/1000 mL. GA, Gargano; SA, Salento; MU, Murgia; JON, Arco Jonico; TAV, Tavoliere.

**Table 2:** Isolation frequency of enteric viruses in 364 water samples from different aquifers.

Virus	Karst-Fissured Aquifers			Porous Aquifers		Total (364) No. (%)	*p*-Value
	GA (28) No. (%)	SA (82) No. (%)	MU (126) No. (%)	JON (40) No. (%)	TAV (88) No. (%)		
Enterovirus	0 (-)	2 (2.4)	0 (-)	1 (2.5)	1 (1.1)	4 (1.1)	0.435
Norovirus	1 (3.6)	3 (3.7)	10 (7.9)	3 (7.5)	1 (1.1)	18 (4.9)	0.194
Rotavirus	1 (3.6)	2 (2.4)	0 (-)	0 (-)	2 (2.3)	5 (1.4)	0.340
Hepatitis A virus	0 (-)	0 (-)	0 (-)	0 (-)	0 (-)	0 (-)	-
TOTAL	2 (7.1)	7 (8.5)	10 (7.9)	4 (10.0)	4 (4.5)	27 (7.4)	0.800

Note: Statistical significance calculated using chi-squared test.

**Table 3:** Wells unsuitable for irrigation use and wells with potential infectious risk owing to the presence of viruses.

Aquifers	Unsuitable Wells */Total Wells No. (%)	Presence of Virus/Suitable Wells * No. (%)	Total Wells with Potential Infectious Risk No. (%)
Karst-Fissured	20/118 (16.9)	17/98 (17.3)	37/118 (31.3)
GA	2/14 (14.3)	2/12 (16.7)	4/14 (28.6)
SA	9/41 (22.0)	6/32 (18.7)	15/41 (36.6)
MU	9/63 (14.3)	9/54 (16.7)	18/63 (28.6)
Porous	15/64 (23.4)	6/49 (12.2)	21/64 (32.8)
JON	3/20 (15.0)	4/17 (23.5)	7/20 (35.0)
TAV	12/44 (27.3)	2/32 (6.3)	14/44 (31.8)
Total	35/182 (19.2)	23/147 (15.6)	58/182 (31.9)

* According to DM 185/03 standards.

**Table 4:** Frequency of positive microbiological results from 182 wells analyzed in winter and summer.

Microbiological Parameters	Winter		Summer		*p*-Value
	No.	%	No.	%	
Total coliforms	113	62.1	118	64.8	0.586
*E. coli*	69	37.9	74	40.7	0.592
Enterococci	73	40.1	79	43.4	0.524
*Salmonella* spp.	3	1.6	7	3.8	0.200
Enteric viruses	15	8.2	12	6.6	0.549

Note: Statistical significance calculated using chi-squared test.
